# Dear Sepsis-3, we are sorry to say that we don't like you*

**DOI:** 10.5935/0103-507X.20170002

**Published:** 2017

**Authors:** António Henriques Carneiro, Pedro Póvoa, José Andrade Gomes

**Affiliations:** 1 Departamento de Medicina, Urgência e Cuidados Intensivos, Hospital da Luz Arrábida - Gaia, Portugal.; 2 Unidade de Cuidados Intensivos Polivalente, Hospital de São Francisco Xavier, Centro Hospitalar de Lisboa Ocidental - Lisboa, Portugal.; 3 NOVA Medical School, Faculdade de Ciências Médicas, Universidade Nova de Lisboa - Lisboa, Portugal.; 4 Unidade de Cuidados Intensivos, Hospital da Luz - Lisboa, Portugal.

On February 23^rd^, 2016, the Journal of the American Medical Association (JAMA) published a proposal for new definitions and criteria for sepsis, which the authors called Sepsis-3.^(1)^ At the same time, the authors named the previous sepsis definitions Sepsis-1 (from 1991)^(2)^ and Sepsis-2 (from 2001)^(3)^ (Table 1). The proposal was prepared by a task force appointed by the European Society of Intensive Care Medicine (ESICM) and the Society of Critical Care Medicine (SCCM), which was composed of 19 specialists in intensive care, infectious diseases, surgery and pneumology. The document was subscribed by 32 scientific societies.^(1)^

**Table 1:** Sepsis-1^(2)^ and Sepsis-2^(3)^ criteria

Sepsis-1	
Sepsis is a systemic inflammatory response in the presence of infection	
SIRS criteria	
Temperature > 38°C or < 36°C	
Heart rate > 90/minute	
Respiratory rate > 20/minute (or PaCO_2_ < 32 mmHg)	
WBC > 12,000/µL or < 4,000/µL (or > 10% immature bands)	
**Sepsis-2**	
General signs and symptoms	Hemodynamic variables
Fever (central temperature > 38.3°C)	Arterial hypotension (systolic < 90 mmHg, MAP < 70 mmHg, or systolic reduction > 40 mmHg in adults or < 2 SD of the normal value for age)
Hypothermia (central temperature < 36°C)	SvO_2_ < 70%
Heart rate > 90/minute or > 2 SD above the normal value for age	Cardiac index > 3.5 L/min/m^2^
Tachypnea	Indicators of organ dysfunction
Edema or positive fluid balance (> 20 mL/kg 24 hours)	Arterial hypoxemia (PaO_2_/FiO_2_ < 300)
Hyperglycemia (glycemia > 120 mg/dL) in the absence of diabetes	Abnormal state of consciousness
Inflammation markers	Acute oliguria (urine output < 0.5 mL/kg/hour)
Leukocytosis (> 12,000/µL) or leukopenia (< 4,000/µL)	Elevated creatinine > 0.5 mg/dL
Normal leukocytes but > 10% immature bands	Coagulation disorders (INR > 1.5/aPTT > 60 s)
Serum C-reactive protein > 2 SD above the normal value	Thrombocytopenia (< 100,000/µL)
Plasma procalcitonin > 2 SD above the normal value	Hyperbilirubinemia (> 4 mg/dL or 70 mmol/L)
	Indicators of tissue perfusion
	Hyperlactatemia (> 1 mmol/L)
	Reduced capillary refill and mottled skin

SIRS - systemic inflammatory response syndrome; PaCO_2_ - partial pressure of carbon dioxide; WBC - white blood cells; SD - standard deviation; MAP - mean arterial pressure; SvO_2_ - venous oxygen saturation; PaO_2_/FiO_2_ - partial pressure of oxygen/fraction of inspired oxygen; INR - international normalized ratio; aPTT - activated partial prothrombin time.

Sepsis became defined as a "life-threatening organ dysfunction caused by a dysregulated host response to infection."

The method used to prepare the proposal was a retrospective analysis of large hospital databases from two countries (the United States and Germany, with considerable predominance of the former) in the attempt to establish the clinical and laboratory parameters that best correlated with mortality among patients with suspected infection.

To identify this cohort of patients with suspected infection in large hospital databases, the authors used non-validated criteria, including patients treated with antibiotics within 72 hours after collection of biological samples for microbiological analysis or patients subjected to sample collection up to 24 hours after the onset of antibiotic treatment.

Because the definition of sepsis came to be centered on "organ dysfunction", the task force suggested using a score of organ dysfunction/failure [i.e., the Sequential Organ Failure Assessment (SOFA)]^(4)^ as the diagnostic criterion for sepsis. According to this suggestion, a patient with an acute change in the SOFA score ≥ 2 meets the criteria for sepsis (Table 2). The task force established that the baseline SOFA score should be zero unless the patient was known to have preexisting (acute or chronic) organ dysfunction before the onset of infection.

**Table 2:** Sequential Organ Failure Assessment (SOFA) score^(4)^

Score	1	2	3	4
Respiratory system (PaO_2_/FiO_2_ - mmHg)	< 400	< 300	< 200 (and ventilation support)	< 100 (and ventilation support)
Coagulation (platelets x 10^3^/mm^3^)	< 150	< 100	< 50	< 20
Liver (bilirubin - mg/dL)	1.2 - 1.9	2.0 - 5.9	6.0 - 11.9	> 12.0
Cardiovascular system (arterial hypotension)*	MAP < 70mmHg	Dopamine ≤ 5 or dobutamine (any dose)	Dopamine > 5 or epinephrine ≤ 0.1 or norepinephrine ≤ 0.1	Dopamine > 15 or epinephrine > 0.1. or norepinephrine > 0.1
Central nervous system (Glasgow Coma Scale)	13 - 14	10 - 12	6 - 9	< 6
Kidneys (creatinine - mg/dL) or urine output - mL/day	1.2 - 1.9	2.0 - 3.4	3.5 - 4.9 or < 500mL/day	> 5.0 or < 200mL/day

* Adrenergic agents must be administered for ≥ 1 hour; doses are expressed as µg/kg/minute; PaO_2_/FiO_2_ - partial pressure of oxygen/fraction of inspired oxygen; MAP - mean arterial pressure.

However, due to the limitations of SOFA outside the intensive care unit (ICU), the task force recommended a new score [i.e., "quick SOFA" (qSOFA)]. This instrument, which was also developed by the task force and was not validated in clinical practice, comprised three clinical parameters that were easy to assess (Table 3) and were associated with high mortality when at least two of them were simultaneously present. In contrast, SOFA includes laboratory data and therapeutic approaches that have different scores according to pre-defined thresholds.

**Table 3:** Sepsis-3 criteria

qSOFA	Septic shock
Respiratory rate ≥ 22/minute Systolic arterial pressure ≤ 100mmHg Altered mentation	Arterial hypotension requiring vasopressors to maintain mean arterial pressure ≥ 65mmHg and hyperlactatemia > 18mg/dL (2mmol/L) despite adequate vascular filling

qSOFA - quick Sequential Organ Failure Assessment.

In turn, septic shock was defined as a "subset of sepsis in which underlying circulatory and cellular metabolism abnormalities are profound enough to substantially increase mortality."

The identification of patients with this condition followed another method and used the Surviving Sepsis Campaign database (28,150 patients from 218 hospitals in 18 countries); this method employed the Sepsis-2 definitions and clinical criteria for infection. The external validation was based on data from two large American hospitals. The criteria for septic shock became a cumulative presence of arterial hypotension (defined as the use of vasopressors) and hyperlactatemia (> 18mg/dL or 2mmol/L) despite adequate volume resuscitation (Table 3).

We emphasize that the category "severe sepsis" was eliminated, which according to the previous criteria characterized septic patients with organ dysfunction and manifestations of hypoperfusion or arterial hypotension associated with sepsis that in prognostic terms had a mortality rate intermediate between sepsis and septic shock.

## The controversy

The medical community became divided over the clinical value of the new criteria (i.e., regarding their actual impact and safety when applied at the bedside). The criticism mainly focused on the following three aspects: (1) underlying theoretical concepts; (2) the methods used to define the criteria; and (3) their potential impacts on clinical practice.

Regarding the theoretical aspects, the criticism emphasized the oddity of applying different criteria to the suspicion and identification of the same pathological phenomenon, which frequently exhibited the same clinical presentation, according to whether or not the patient was admitted to the ICU. The criticism stressed that the new criteria stemmed from a purely retrospective analysis of hospital databases created for completely different purposes, were quite limited in their geographic distribution, and defined for this particular objective, infection (i.e., a clinical concept) as a "collection of biological samples + prescription of antibiotics within a given time interval" (i.e., non-clinical concepts) and using physiological data collected in a manner that was not completely explained (i.e., the reliability of the Glasgow Coma Scale assessment or the respiratory rate, especially outside the ICU). Clearly, this criticism only applies to the development of the criteria for sepsis and not to the criteria for septic shock, which as mentioned above are based on another set of data.

Without downplaying the relevance of the first two aspects, we believe that the future use of these criteria in clinical practice (i.e., the potential clinical impact of their application at the bedside) is a cause of great concern. The Sepsis-3 criteria introduce no changes in the approach to sepsis, especially concerning antibiotic treatment, fluid therapy, and vasopressor support, but neglect the early identification of sepsis before the development of organ failure.

## Relationship between the Sepsis-1 and Sepsis-2 criteria and the new Sepsis-3 criteria

**Figure 1:**
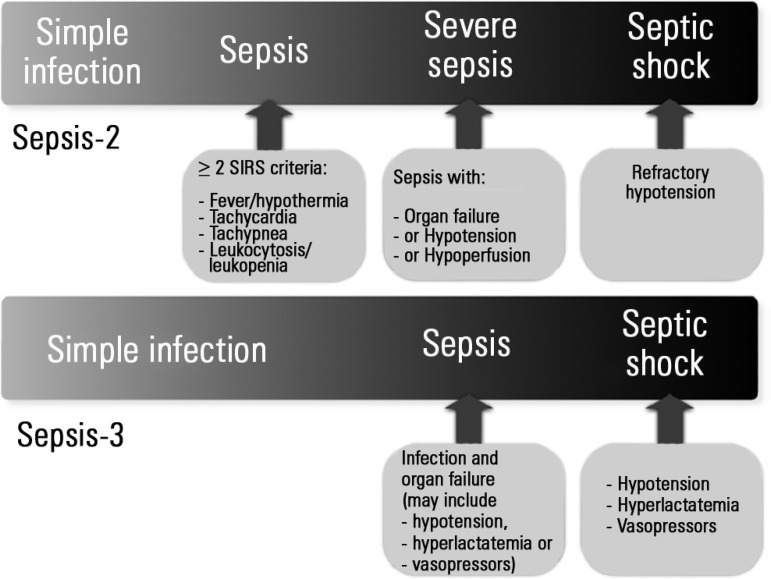
Relationship between the Sepsis-2 and Sepsis-3 classifications. SIRS - systemic inflammatory response syndrome.

Following the Sepsis-3 criteria, the previous categorizations of the severity and consequent mortality due to infection that progressed from sepsis (infection meeting the criteria for systemic inflammatory response syndrome or SIRS) to severe sepsis (sepsis with organ failure, arterial hypertension, and/or hypoperfusion) to septic shock (arterial hypotension refractory to adequate volume resuscitation) were reduced to simple infection, sepsis (infection and manifestations of organ failure), and septic shock (arterial hypotension defined as the use of vasopressors and hyperlactatemia) (Figure 1). Sepsis-2 concept of severe sepsis roughly corresponds to the definition of sepsis in the Sepsis-3 criteria, although this correlation is not absolute because sepsis, according to the new criteria, can include very different conditions, such as organ failure without hypotension nor hyperlactatemia, arterial hypotension even when vasopressors are used in any dose provided the lactate level is ≤ 18mg/dL (2mmol/L; i.e., vasoplegic shock), and also cryptic shock (hyperlactatemia without hypotension).^(5-8)^

The combination of the blood pressure values (or use of vasopressors after adequate volume resuscitation) and lactatemia has been long known to allow the identification of patients with different severities and prognoses, which to date were systematized as follows:

1. Cryptic shock: defined as lactate concentration ≥ 4 mmol/L without arterial hypotension (or use of vasopressors).
2. Septic shock: hypotension induced by sepsis that persisted despite adequate volume resuscitation and might present as:
  2.1. Vasoplegic shock: hypotension refractory to fluid therapy with normal serum lactate.
  2.2. Shock with tissue dysoxia: hypotension refractory to fluid therapy with hyperlactatemia.

The criteria defining the last group, which exhibits higher mortality, are the criteria the authors of Sepsis-3 considered necessary for the definition of septic shock. In other words, the various phenotypic expressions of the severity of septic shock are not considered in Sepsis-3, because only dysoxic septic shock is taken into account and the vasoplegic and cryptic shock categories are ignored. The latter categories were classified as sepsis.

Another major issue is whether we can undervalue the relevance of clinical manifestations that, according to the Task Force, are now called "infection" and that until recently were septic patients with different morbidities and mortalities. Besides, the mortality of these conditions is not negligible, as may be inferred from the tables published by the authors of the Sepsis-3 manuscript themselves.^(9)^

## Do we need new criteria for sepsis?

As in every other situation, any change made should have a purpose. Are the previous criteria less useful and restrictive for the management of more severe infections? The clinical evidence points to the opposite situation. The ultimate goal of our action as physicians is to reduce morbidity and mortality.

The criteria for SIRS were the target of much criticism for having too high sensitivity but poor specificity. In turn, the term "severe sepsis", with its consequent organ dysfunction and/or tissue hypoperfusion and/or arterial hypotension associated with sepsis, was considered by several researchers (namely, the developers of Sepsis-3) to be the true onset of septic conditions.

From our perspective, the approach to sepsis should be grounded on three fundamental aspects that should be considered simultaneously and based on demonstrated proof for its management and treatment as follows: (1) early recognition and stratification of severity; (2) prevention of and support for organ dysfunction based on an optimal oxygen delivery; and (3) treatment of the cause and control of the infection site.

To attain these goals, the Surviving Sepsis Campaign (SSC) sets of documents are available. These documents contain recommendations that indicate standardized, goal-oriented diagnostic and therapeutic actions according to the patient's severity and response to treatment based on early identification and stratification of sepsis patients (Sepsis-2). These recommendations were updated every four years, with the latest version published in 2013.^(10)^ Admittedly, the Sepsis-2/SSC partnership has an optimal record of success,^(7,11,12)^ with a significant impact on mortality by doing more with the available resources (i.e., without any new medication).

## How should we ground our action in the face of infection?

We should always keep in mind that there is no pathophysiological aspect that is pathognomonic of sepsis and that the diagnosis of infection results from the intersection of three vectors (systemic manifestations, manifestations of organ dysfunction, and microbiological documentation), because no specific marker is known at present.

In reality, we do not know whether the Sepsis-2 or the Sepsis-3 criteria best identify the most severe cases of infection that demand more timely therapeutic management. However, we fear that downplaying infectious conditions that do not meet the current Sepsis-3 criteria (i.e., the earliest cases and cases that have a less severe presentation) will hinder their identification, resulting in an unnecessary increase in both morbidity and mortality due to their inexorable progression in the following hours. We admit that this risk is purely theoretical at present.

We anticipate that studies comparing the performance of both criteria in the real world will be conducted in the near future. Independent from their results, our approach to the patient with suspected infection should always be clinical. We should strive to achieve the identification of the initial and sometimes subtle manifestations of organ failure and hypoperfusion in all patients with suspected infection; however, these manifestations are devalued in the Sepsis-3 criteria in favor of scores (SOFA and qSOFA).

Although not formally validated, the various criteria included in Sepsis-2 have extraordinarily high sensitivity for the early stratification of infection. When these criteria are followed by the application of the SSC recommendations, they have an impressive history of success in reducing the mortality of sepsis in several areas of the world.^(5,11-13)^ The authors of Sepsis-3 conclude their text by asserting, "These updated definitions and clinical criteria should clarify long-used descriptors and facilitate earlier recognition and more timely management of patients with sepsis or at risk of developing it." Unfortunately, our perception suggests the opposite outcome. SSC warns against this same risk by asserting, "The following advice is meant to put the recent publication of the consensus definitions in context to facilitate the continued successes of sepsis screening, early identification and treatment that have been the hallmark of SSC's quality improvement efforts associated with improved survival during the preceding decade".^(14)^
